# Psychoanalytically informed MDMA-assisted therapy for pathological narcissism: a novel theoretical approach

**DOI:** 10.3389/fpsyt.2025.1529427

**Published:** 2025-04-02

**Authors:** Alexa E. Albert, Anthony L. Back

**Affiliations:** ^1^ Department of Psychiatry and Behavioral Sciences, University of Washington School of Medicine, Seattle, WA, United States; ^2^ Seattle Psychoanalytic Society and Institute, Seattle, WA, United States; ^3^ Department of Medicine, University of Washington School of Medicine, Seattle, WA, United States

**Keywords:** 3,4-methylenedioxymethamphetamine (MDMA), pathological narcissism (PN), narcissistic personality disorder (NPD), personality disorders, psychedelics, psychoanalytic, early relational trauma, developmental trauma

## Abstract

Pathological narcissism (PN) is a complex, treatment-resistant disorder characterized by unstable self-esteem that fluctuates between grandiosity and vulnerability, complicating the formation of a stable self-image. With few empirically supported therapies, treatment has traditionally relied on long-term psychoanalytic approaches, but these often face high attrition. Recent research suggests a potential therapeutic synergy between psychedelics and psychoanalytic therapy, offering a novel approach to addressing entrenched personality structures. Studies on MDMA (3,4-methylenedioxymethamphetamine), a compound known for enhancing empathy, trust, and social interactions, demonstrate potential to reopen critical periods for social learning in adults, offering the possibility of therapeutic benefits for conditions with core issues in relatedness, such as PN. MDMA promotes psychological flexibility and openness, allowing for deeper self-exploration and strengthening the observing ego, considered in psychoanalytic therapy to be an essential component for recognizing and modifying maladaptive patterns. By reducing fear-based avoidance in the brain, MDMA facilitates access to unconscious emotions, helping individuals process overwhelming feelings linked to early relational trauma commonly seen in PN. Additionally, MDMA’s capacity to enhance compassion and empathy can fortify the therapeutic alliance, increasing its potential to facilitate relational change. This paper presents an MDMA-assisted therapy (MDMA-AT) tailored for narcissistic patients which is currently being conducted as an investigator-initiated trial (IIT). It explores the model’s theoretical foundations, mechanisms of change, treatment framework, and clinical challenges. Combining MDMA with an evidence-based depth therapy like psychoanalytic psychotherapy may offer an innovative treatment for conditions associated with attachment and developmental trauma, particularly personality disorders. While the role of psychotherapy in psychedelic treatments remains a topic of debate, with some proposing psychedelics be administered without psychotherapy, we assert that individuals with early relational trauma stand the most to gain from an integrated psychedelic-assisted therapy (PAT) model, where MDMA enhances the therapeutic alliance and emotional openness while psychoanalytic interventions provide the structure for lasting change.

## 1 Introduction

Emerging research supports the safety and efficacy of psychedelic-assisted therapy (PAT) when administered in a controlled clinical environment (1, 2) for several treatment-resistant psychiatric disorders including depression (3–5), post-traumatic stress disorder (PTSD) (6, 7), substance use disorders (8, 9), social anxiety (10), and obsessive-compulsive disorder (11). However, the potential for psychedelics to treat personality disorders remains unclear, as most clinical studies on PAT have explicitly excluded individuals with these conditions. Thus, a scientific gap exists around the potential for psychedelic therapies to treat personality disorders. Little evidence exists to assess the range of psychedelic effects, safety considerations, and applicability of treatment for these patients (12, 13). Expanding research to include individuals with personality disorders could provide further insight into the generalizability and mechanisms of PAT.

Personality disorders are characterized by rigid patterns of thinking, feeling, and behaving that deviate from what is considered normal or expected in an individual’s particular culture. Individuals with personality disorders have difficulty responding flexibly and adaptively to the demands of life, and their rigid ways of responding often perpetuate and intensify their difficulties. Fundamental problems in *relatedness* span all personality disorders, specifically difficulties in achieving a stable, positive sense of self and establishing enduring, mutually gratifying relationships (14, 15). While the precise etiology of personality disorders remains uncertain, research suggests they result from a complex interaction between genetic, environmental, epigenetic, and developmental factors, with childhood mistreatment strongly linked to subsequent impairments in both intrapersonal and interpersonal functioning (16).

Concerns raised by naturalistic studies and trial design complexities have contributed to the exclusion of personality disorder populations from psychedelic research. One naturalistic, uncontrolled study found that individuals with personality disorders were over four times more likely to experience worsened mental health following psychedelic use of psilocybin, lysergic acid diethylamide (LSD)), ayahuasca, N,N-Dimethyltryptamine (DMT)), mescaline, and iboga (17). Additional reported concerns include the risk of emotional instability during sessions, challenges to establishing therapeutic rapport, and difficulty integrating psychedelic experiences (17–19). Researchers also report complexity standardizing protocols and analyzing results in this population (20). Moreover, comorbid conditions such as suicidality and substance use disorders further complicate research efforts (13, 18, 20, 21). These concerns, along with historical caution stemming from early psychedelic research, have reinforced the exclusion of personality disorders from clinical trials (22, 23).

In spite of these challenges, interest in PAT for personality disorders is increasing (24, 25). With longstanding funding priorities having favored mood and psychotic disorders (14), innovation in personality disorder treatments has been limited, with the notable exception of novel therapies for borderline personality disorder (26–30). However, researchers have recently suggested psychedelics as potential treatment based on evidence that psychedelic therapy can change patterns of cognitive-affective rigidity, emotion dysregulation, and interpersonal dysfunction (31, 32).

In the few recent studies that measured personality traits, psychedelics were linked to reductions in neuroticism and increases in openness and extraversion across both non-clinical and clinical populations (33, 34). Studies using the five-factor model of personality suggest that psilocybin increases openness in healthy participants (34) and enhances extraversion in clinical populations with alcohol use disorder, with some early evidence also pointing to potential increases in conscientiousness, though this finding was not statistically reliable (35). Additionally, psilocybin therapy has been associated with sustained decreases in neuroticism among individuals with treatment-resistant depression (33) and alcohol use disorder (35). While the exact mechanisms of action remain unclear, and despite study limitations including small sample sizes and challenges in maintaining blindness in placebo-controlled trials (36), these preliminary findings support the potential use of psychedelics in personality disorder treatment (18, 25, 37, 38).

Among the personality disorders, pathological narcissism (PN) - a broader construct that includes but is not limited to the diagnostic criteria for narcissistic personality disorder (NPD) - stands out as a candidate for evaluating the efficacy of psychedelic therapy. One study prospectively examined changes in grandiose narcissistic personality traits and antagonistic externalizing behaviors in adults following ayahuasca ceremonies, finding modest reductions in entitlement and increases in leadership authority up to three months post-retreat, though effect sizes were small and results mixed across measures (39). In a feasibility study of MDMA therapy for borderline personality disorder (BPD), Inouye et al. (37) raised the possibility of psychedelic therapy for NPD, citing research conducted in Switzerland from 1988 to 1993 under special government permission, using low-to-moderate dose MDMA and LSD during therapy on an undisclosed number of patients with NPD, without any reports of significant or severe negative side effects (40, 41).

Like BPD, PN is a compelling candidate for PAT due to its prevalence, associated impairments, and resistance to conventional treatments. While difficulties in self-concept stability, emotion regulation, and interpersonal functioning are common across personality disorders, PN presents a distinct profile of these deficits that MDMA’s therapeutic effects may be particularly suited to address. With actions that enhance these very domains, MDMA represents a novel potential treatment for PN. This hypothesis paper will first examine the conceptualization, development, neurobiology, and treatment of PN and then discuss a novel MDMA-assisted therapy (MDMA-AT) protocol that integrates psychoanalytic principles and is currently being evaluated in a pilot study.

## 2 Pathological narcissism across development, neurobiology, and treatment

The term PN has been increasingly adopted over NPD, as it encompasses both grandiose and vulnerable expressions of narcissistic dysfunction (42). Unlike the DSM-5 conceptualization of NPD (43), which primarily emphasizes grandiosity, PN accounts for fluctuations in self-regulation and interpersonal dynamics, offering a dimensional rather than categorical perspective on narcissistic pathology. This broader framework better aligns with clinical reality and empirical evidence, and provides a more comprehensive understanding of narcissistic dysfunction. Previously regarded as a rare and obscure psychological disorder (43), PN is now recognized as a relatively common personality disorder, with prevalence estimates in the U.S. general population reaching up to 6.2% (44–46), with notably higher rates among men (7.7%) than women (4.8%) (44).^1^

Strongly associated with a history of child maltreatment, particularly neglect (47–49), PN is believed to develop in response to early traumatic empathic failures by caregivers who were experienced as “confusing, unpredictable, and full of hidden agendas” (50). Inconsistent and untrustworthy caregiving fosters insecurity and instability in a child’s sense of self, resulting in a fragile self-esteem that becomes heavily reliant on external affirmation (51, 52). To manage deep-seated needs for admiration and intense yearnings for approval, the child learns to present a false self, masking true feelings and vulnerabilities that have been unrecognized or deemed unacceptable by their caregivers (53). This adaptation defends against rejection while it also heightens sensitivity to exposure, criticism, and disapproval.

Dissociation is a key factor in the development of PN, especially among those individuals who have experienced childhood maltreatment. Research indicates that betrayal trauma can lead to dissociative coping strategies in those with narcissistic traits (52), and studies suggest that dissociation mediates the relationship between early maltreatment and both grandiose and vulnerable expressions of narcissism (49). These findings suggest that dissociation functions as a means of coping with early emotional neglect and instability, and is perhaps at the root of the shifting self-states seen in PN. The affective experience of fundamentally not being seen or known by one’s caregivers during critical developmental periods alters a child’s sense of self, affecting their sense of self-worth and agency as well as impairing their ability to form secure attachments, often resulting in the divestiture of meaningful emotional investment in themselves and others.

PN is characterized by fundamental instability in self-esteem regulation, leading to dynamically alternating states of grandiosity and vulnerability (54). Narcissistic vulnerability manifests as a brittle sense of self, emotional dysregulation and reactivity, and impaired empathic functioning, coupled with excruciating feelings of shame, self-doubt, contempt, envy, and loneliness. To counter these vulnerabilities, individuals employ maladaptive, self-enhancing strategies to bolster a view of themselves as exceptional and deflect recognition of their vulnerabilities (55). Recent research has confirmed the clinical validity of this model, reinforcing its relevance in understanding narcissistic pathology (56). Empirical evidence supports this conceptualization, demonstrating that narcissistic individuals exhibit high explicit but fragile implicit self-esteem, contributing to self-esteem instability and driving reliance on maladaptive self-enhancement strategies (57). These grandiose expressions often impede meaningful relationships, impairing mutuality and reciprocity. Neuroimaging studies provide evidence for psychoanalytic theories associating narcissistic impairments with early attachment disturbances and disrupted self-development, identifying structural and functional irregularities in brain areas involved in self-regulation and social cognition (58).

With a prevalence of up to 20% in clinical populations (59) and high rates of psychiatric comorbidity - including mood, anxiety, substance use disorders, and other personality disorders (60) - PN remains significantly underdiagnosed and undertreated. This is largely due to stigmatization and limited research focus respectively, with few high-quality population studies and most existing knowledge derived from small sample-size investigations, case reports, and case series (61). The clinical impact of untreated PN extends to the individuals with the condition as well as to those around them - especially their romantic partners and children, who are particularly vulnerable to the transmission of intergenerational trauma (62–65). This has led to a greater awareness of the need for mental health support and treatment for those affected by PN (66–68), bolstered by popular accounts of public figures.

Patients usually do not seek treatment until they are in acute crises, such as personal or professional failures, severe interpersonal conflicts, or increasing dissatisfaction with life. During these crises, symptoms of narcissistic vulnerability are most prominent, including acute suicidality, anhedonic depression, and anxiety (54, 69). Narcissistic grandiosity often only becomes evident post-crisis. Despite high dropout rates of 63-64% (70) psychodynamic therapies remain the conventional treatment for PN, with long-term individual psychotherapy as the cornerstone (71). Evidence-based treatment guidelines for PN have not yet been established. More recently, a few empirically supported treatments originally developed for borderline personality disorder have emerged as adaptations for PN, such as dialectical behavior therapy (DBT) (72), transference-focused psychotherapy (TFP) (73), mentalization-based therapy (MBT) (74, 75), and schema therapy (76). To date, there is no strong empirical evidence supporting pharmacotherapy for PN, with medications primarily used to target comorbid symptoms such as mood instability, impulsivity, and aggression rather than core narcissistic traits (77).

## 3 A psychoanalytically informed MDMA-assisted therapy: a novel approach

Given the challenges of conventional PN treatment - including lengthy treatments, high patient attrition, and limited evidence-based outcomes (70, 78) - we propose a novel treatment approach: a psychoanalytically informed MDMA-assisted therapy (MDMA-AT) tailored for narcissistic patients.^2^ Given preliminary evidence indicating that MDMA can bolster self-confidence, promote emotional openness, reduce fear responses, and increase sociability in clinical settings (1), we hypothesize that combining MDMA with psychoanalytically informed psychotherapy could be particularly efficacious in addressing the social-emotional deficits characteristic of PN. This section outlines the theoretical foundations, mechanisms of change, treatment framework, and clinical challenges of this theoretical approach that is currently under investigation.

### 3.1 Theoretical foundations

#### 3.1.1 Why MDMA?

MDMA (± 3,4-methylenedioxymethamphetamine) is a psychoactive compound with unique pharmacological and therapeutic properties that differentiate it from classical psychedelics like psilocybin and LSD. While classical psychedelics primarily act as 5-HT2A receptor agonists, inducing profound perceptual and cognitive alterations, MDMA primarily stimulates the release of serotonin (5-HT), norepinephrine (NE), and dopamine (DA), while also promoting oxytocin release. This neurochemical profile contributes to its prosocial effects, including heightened emotional openness and trust, reduced fear responses, and increased receptivity to social connection, all while generally preserving cognitive clarity and perceptual stability (1). Due to these therapeutic actions, MDMA is often classified as an *entactogen* (promoting introspection and self-reflection) and an *empathogen* (enhancing empathy and compassion). More recently, the term *connectogen* has been proposed to better capture MDMA’s ability to foster deep, holistic connections that are both intrapersonal (entactogenic) and interpersonal (empathogenic) (79).

MDMA’s primary mechanism involves increasing the availability of 5-HT and NE, while indirectly influencing DA via monoamine transporter activity and triggering oxytocin release (79–82). These neurochemical effects contribute to mood elevation, heightened self-confidence, and greater emotional openness (1, 83). MDMA’s ability to enhance social bonding is thought to be driven by its stimulation of oxytocin release (84), which increases prosocial feelings (85), greater trust in interpersonal interactions (86), and openness to social reward and connectedness (87). Additionally, MDMA promotes a desire for social engagement, potentially by reducing sensitivity to subtle negative emotional cues in others (88). MDMA also appears to modulate fear responses by reducing activity in the amygdala, decreasing reactions to socially threatening stimuli while enhancing responses to socially rewarding signals - an effect that may influence emotional memory processing (89). Moreover, MDMA has been shown to reduce fear responses during trauma recall, likely through its effects on both the amygdala and hippocampus, facilitating memory reconsolidation (90).

MDMA’s ability to enhance global cortical connectivity and neuroplasticity is also believed to play a key role in its therapeutic effects (91). Neuroimaging studies demonstrate that MDMA increases cortical-limbic communication, integrating previously segregated brain networks while reducing rigid connectivity in associative areas - a pattern linked to greater cognitive flexibility and emotional openness (91). Additionally, MDMA reduces activity in the Default Mode Network (DMN), potentially loosening rigid self-referential thinking patterns (92). Given that PN is characterized by inflexible and defensive cognitive processes, MDMA’s capacity to soften entrenched self-concepts may have significant therapeutic implications. MDMA’s influence on the salience network also facilitates a shift toward positive emotional experiences, reinforcing affective learning and emotional regulation (93, 94). This suggests a therapeutic potential for PN, where hypersensitivity to social evaluation contributes to maladaptive interpersonal dynamics. By reducing excessive salience network activity, MDMA may help individuals with narcissistic traits to recalibrate their emotional responses and engage more adaptively in social interactions.

Neuroimaging studies indicate that individuals with high narcissistic traits exhibit reduced grey matter volume in the anterior cingulate cortex (ACC), medial prefrontal cortex (mPFC), and dorsolateral prefrontal cortex (dPFC) - regions critical for self-awareness, emotion regulation, and social cognition (58, 95). Despite these structural differences, individuals with PN display heightened anterior insula (AI) and dorsal ACC (dACC) activity in response to social exclusion, indicating increased rejection sensitivity despite an outward appearance of emotional detachment (96). By enhancing ACC function and promoting cognitive flexibility, emotion regulation, and fear extinction via 5-HT2A receptor activation (97), MDMA may help reduce defensive rigidity and social threat hypersensitivity - core features of PN that contribute to its paradoxical combination of emotional detachment and heightened sensitivity to threats against self-image or social status (98). Additionally, MDMA’s modulation of the amygdala and salience network may attenuate exaggerated threat responses to social evaluation, facilitating more adaptive interpersonal processing (98). By reducing defensive rigidity and enhancing emotional openness, MDMA may help individuals with PN engage more flexibly in social interactions.

MDMA’s ability to modulate neurotransmitters, enhance neuroplasticity, reorganize brain networks, and influence emotional processing ultimately leads to the activation of core emotional circuits and unconscious mechanisms. Clinical studies consistently link psychedelic experiences to increased access to emotions, memories, and perceptions typically avoided or outside conscious awareness (99, 100). How do these neurobiological changes translate into the profound therapeutic shifts observed in PAT? The REBUS (Relaxed Beliefs Under Psychedelics) model, proposed by Carhart-Harris and Friston (101), offers a functional framework for understanding brain dynamics under psychedelics. It suggests that psychedelics “relax” high-level cortical cognitive structures, or “priors” - deep-seated beliefs and assumptions that shape how individuals perceive and interpret the world. These priors often suppress new information to maintain a stable worldview, even when this results in rigid or pathological thinking patterns that contribute to mental illness. Under the influence of psychedelics, the precision of these priors is reduced, allowing bottom-up subcortical sensory information, along with dissociated memories, emotions, and thoughts, to surface. This process creates a therapeutic window during PAT in which entrenched pathological beliefs can be revised, promoting psychological flexibility. This window represents the opening of a critical learning period, that promotes plasticity in thought, emotion, behavior, and interpersonal relationships that lasts for approximately two weeks after an MDMA session (87).

MDMA creates a state of heightened openness and emotional receptivity that extends beyond the acute drug experience, offering a critical window for therapeutic intervention. MDMA’s softening of rigid self-protective mechanisms reduces interpersonal defensiveness, and increases sensitivity to emotional and relational dynamics, allowing for deeper engagement in the therapeutic process (88, 100, 102). These effects are particularly relevant for narcissistic patients whose defense mechanisms often preclude emotional insight and connection. The two-weeks following MDMA administration is associated with increased neuroplasticity and psychological flexibility making it an important time for integration. Given this extended window of time, the structure and approach of psychotherapy during MDMA-AT become central considerations.

#### 3.1.2 Why psychoanalytically informed therapy?

The role of psychotherapy in psychedelic treatment remains a subject of debate (103). Key discussions focus on whether psychotherapy should accompany psychedelic use and, if so, the optimal amount and most efficacious approach. Some argue that psychedelics could serve as standalone treatments, with psychological support primarily serving a safety function (104). Others highlight the scalability and cost-effectiveness of PAT, advocating for research into shorter, more affordable treatment schedules (105).

Conversely, proponents of a synergistic approach contend that psychedelics enhance therapeutic outcomes when combined with psychotherapy (106–109). Debate continues over which therapeutic modalities best complement psychedelic treatment (110–112), with various adapted and *ad hoc* models being evaluated in clinical research presently (106). Further research is needed to identify the most efficacious therapeutic modalities that synergize with psychedelics to optimize clinical outcomes (103). Clarifying which therapeutic modalities best complement psychedelic treatment remains a key priority for future research (103).

Amid these ongoing debates, the absence of clinical trials exploring psychoanalytic approaches to PAT is particularly notable. Despite substantial evidence supporting the efficacy of psychoanalytic modalities, research has largely overlooked their integration with psychedelic therapy as well as their compatibility. Importantly, there appears to be growing support among psychoanalysts as well as psychiatrists for psychedelic-assisted therapies for their patients (113, 114). Unlike the more manualized treatments, psychoanalytic therapy is inherently open-ended, following and supporting a patient’s largely self-directed process. This flexibility makes it particularly compatible with psychedelics, whose effects are often unpredictable and require therapists to adapt to patients’ evolving processes (103). Moreover, although they operate on different timescales, psychoanalytic therapy and psychedelics share a remarkable therapeutic convergence. Both approaches are believed to activate the brain’s core emotional systems and unconscious processes to result in psychological change.

Contemporary psychoanalytic theory posits that psychological disorders arise from failures to meet innate emotional needs (115).^3^ Neurobiological findings align with key psychoanalytic concepts, particularly regarding instinctive needs, unconscious processing, and the centrality of affect in mental life (115, 116). These findings suggest that feelings originate in subcortical structures and serve as expressions of basic unmet emotional needs and motivations. Unlike physiological symptoms, psychological ones carry meaning and intention, reflecting the patient’s struggle to satisfy these needs (e.g., panic arises from fear of losing an attachment, while rage occurs when the satisfaction of one’s needs is frustrated). Deeply rooted in early life experiences, coping behaviors become unconscious automatic responses or “automatized predictions” (115) over time. Developed to fulfill unmet needs and based on learned experiences from the past, these unconscious patterns of behavior are not consciously remembered but are automatically re-enacted – that is, replayed in current relationships or situations as an attempt to gain mastery over or integrate unresolved psychological conflicts (117, 118). In short, both psychoanalytic and psychedelic therapy facilitate access to the unconscious to revise unconscious processes (i.e., “automatized predictions” per psychoanalytic theory and “priors” per the REBUS model), adapt emotional regulation, and rework patterns of relating to oneself and others, ultimately driving personality transformation.

Psychoanalytic treatment helps patients recognize and work through unconscious conflicts that shape their emotions and behaviors. By bringing unconscious coping mechanisms into awareness, it enables individuals to break maladaptive patterns, address unmet needs, and develop healthier ways of regulating emotions. Psychoanalytic therapy has demonstrated efficacy comparable to other evidence-based treatments, such as cognitive-behavioral therapy (CBT), while remaining distinct in its emphasis on early development and unconscious processes (119). Its effect sizes range from 0.78 to 1.46, comparable to or even exceeding those of other evidence-based treatments (120, 121). Comparison studies of depression treatments indicate that psychoanalytic psychotherapies emphasizing interpersonal and developmental experiences lead to both short- and long-term improvement, with a developmental focus predicting longer recovery and better functioning over 24 months than other therapies (122). Research indicates that combining psychoanalytic techniques with cognitive therapy enhances treatment outcomes, whereas cognitive techniques alone demonstrate limited association with long-term improvements (123).

Its distinctive focus on increasing awareness of unconscious feelings, beliefs, and reflexive coping patterns - rather than just symptom relief - may explain why psychoanalytic therapy is particularly efficacious for facilitating lasting transformation compared to cognitive-behavioral approaches (124). Research suggests that the benefits of psychoanalytic therapy not only endure, but its effect size may actually increase over time, reaching up to 1.51 at long-term follow-up (120, 121). This phenomenon, known as the “sleeper effect,” implies that psychoanalytic therapy initiates ongoing processes of change that continue to evolve even after therapy has ended (121).

With the unique characteristics of psychoanalytic therapy empirically distinguished from other treatment methods (125), neuropsychoanalysis pioneer Solms (115) has identified key psychoanalytic techniques that align with neuroscientific findings and are associated with optimal treatment outcomes. These techniques are thought to engage the brain’s deeper emotion-driven systems, facilitating long-term psychological change. Notably, while these same techniques are fundamental to psychoanalytic therapy, research suggests they also contribute to effective outcomes across various therapeutic orientations, including CBT, even when their influence is not explicitly recognized (121):

1. Facilitating “unstructured, open-ended dialogue between patient and therapist.
2. Identifying recurring themes in the patient’s experience.
3. Linking the patient’s feelings and perceptions to past experiences.
4. Drawing attention to feelings regarded by the patient as unacceptable.
5. Pointing out ways in which the patient avoids feelings.
6. Focusing on the here-and-now therapy relationship.
7. Drawing connections between the therapy relationship and other relationships” (115).

With its attention on the here-and-now, psychoanalytic therapy is uniquely suited to PAT. Zamaria et al. (103) describe PAT as inherently relational, which is in keeping with the contemporary ethos of psychoanalytic therapy, where the importance of the therapist-patient dynamic helps facilitate change. Attending to the therapy relationship often reveals unconscious conflicts, particularly through transference, which involves patients projecting past emotions and expectations onto the therapist. These projections are largely shaped by early childhood attachment experiences and interpersonal patterns, reflecting deeply ingrained relational templates (14, 115, 126). Countertransference, in turn, refers to the therapist’s emotional reactions, often influenced by their own unresolved conflicts. Once made conscious, these unconscious patterns offer key insights into relational dynamics and can serve as powerful tools for fostering self-understanding and transformation.

With its focus on uncovering entrenched intra- and interpersonal dynamics, psychoanalytic therapy offers a structured framework for treating personality disorders, particularly PN, where rigid behavioral defenses often impede progress. While psychoanalytic therapy facilitates personality restructuring, MDMA-assisted therapy enhances emotional openness and access to unconscious material. However, their combined potential remains unexplored. By integrating their complementary mechanisms, we propose a novel framework to optimize therapeutic outcomes for PN.

### 3.2 Hypothesized mechanisms of change

To operationalize this integration, we examine the mechanisms through which MDMA may enhance psychoanalytic interventions and facilitate change in the treatment of PN. MDMA’s effects - heightened emotional openness, reduced fear, and increased sociability - may soften defense mechanisms that obstruct psychoanalytic exploration. By temporarily weakening rigid self-protective patterns, MDMA can deepen engagement, allowing psychoanalytic interventions to more effectively address core personality dynamics and increase receptivity to therapeutic insights. Psychedelics have been shown to enhance psychotherapeutic outcomes by facilitating cognitive, emotional, and behavioral transformations (103, 106, 127). We hypothesize that MDMA will augment psychoanalytic therapy and influence the cognitive-affective rigidity, emotion dysregulation, and interpersonal dysfunction characteristic of PN. The specific mechanisms through which these effects occur will be measurable in our pilot study.

#### 3.2.1 Indicators of change in narcissistic patients

Core indicators of change in PN identified by Ronningstam and Weinberg (78) will be utilized to measure treatment efficacy. Their research importantly demonstrates that patients with PN are capable of change (78) and that meaningful therapeutic progress is linked to improvements in reflective functioning and self-awareness, emotion regulation and tolerance, and a strengthened sense of agency (78). They also emphasize that a strong therapeutic alliance can foster self-reflection and emotion regulation in patients with PN, while increasing awareness of their internal experiences and interpersonal impact. In turn, these effects may reduce defensiveness and enhance empathy and self-compassion (128), further supporting therapeutic change.

Changes in personality pathology and functioning are best understood as a multifactorial, integrative process, in which various factors, individual characteristics, and contextual influences can either facilitate or hinder a patient’s capacity for change. Ronningstam and Weinberg (78) found that therapeutic interventions promoting self-reflection, agency, and openness to new experiences were essential for patients with PN, from the first therapeutic session onward. By operationalizing these indicators as treatment outcomes, our study introduces MDMA as a novel factor that may enhance patient openness and facilitate engagement with the therapeutic process. If MDMA-AT promotes greater receptivity to self-examination and emotional processing, it could play a significant role in overcoming the psychological barriers that often impede change in PN and support its potential as an intervention for narcissistic pathology.

#### 3.2.2 Reflective ability and self-awareness

Reflective functioning, or mentalization, is the ability to understand one’s own and others’ thoughts and feelings (129). It is essential for self-regulation and interpersonal functioning, enabling individuals to process emotions, adjust maladaptive patterns, and build healthier relationships. Deficits in mentalization are linked to personality disorders, including PN, where individuals often engage in intellectualized, detached rumination rather than genuine reflection (129, 130). Enhanced reflective capacity is a key mechanism of change in personality disorder treatment (131). Ronningstam and Weinberg (78) highlight reflective functioning as central to change in narcissistic individuals, facilitating a shift from blame and avoidance to responsibility and collaboration. Mentalization-based therapy (MBT) has been adapted to address these deficits in PN, with theoretical and clinical support suggesting it promotes a transition from a rigid, self-focused “me-mode” to a more relational “we-mode,” leading to greater self-reflection, emotional awareness, and perspective-taking (132). Improvement in reflective ability in PN not only signals change but also contributes to better emotion regulation, increased self-agency, and stronger therapeutic engagement (78).

In the 1970s, MDMA was used in psychotherapy to enhance self-awareness (79). Among recreational MDMA users, MDMA use has been linked to increased self-compassion and reduced self-criticism post-use (133). Research shows MDMA increases authenticity, self-acceptance, and introspection, all supporting reflective functioning (79). MDMA has been shown to improve psychological flexibility, reduce fear of negative evaluation, and enhance self-reflection (89, 134–136). Additionally, MDMA has demonstrated it enhances metacognitive capacity, promoting self-other differentiation and deeper emotional processing, both of which are crucial in working with narcissistic pathology (137). Its prosocial effects foster emotional openness and increased social cognition (138, 139). These findings suggest MDMA could help narcissistic patients engage more deeply in self-reflective processes necessary for therapeutic change.

Blay et al. (140) provide evidence that impaired self-mentalization drives emotion dysregulation in narcissistic individuals, making both key treatment targets. MDMA facilitates introspection and emotional integration (137, 138). While research on MDMA’s effects on mentalization in structured therapy is limited, its observed impact on self-reflection and emotional processing suggests potential for further study. Given that mentalization deficits contribute to personality pathology (141), investigation is warranted to assess MDMA’s role in enhancing reflective functioning in PN.

#### 3.2.3 Emotion regulation and tolerance

Developing emotional tolerance and regulation is essential for therapeutic progress in narcissistic individuals (78, 128). Patients with PN struggle to access, recognize, and articulate their emotional experiences (60). Emotion regulation involves integrating painful experiences, mourning losses, and accepting personal limitations. Psychoanalytic theory has long emphasized grief as central to emotional development and mental health (142). Bowlby (143) identified mourning as crucial for adaptation, while Kohut (144) linked narcissistic injuries to unmet childhood needs, necessitating the grieving of idealized self and other representations. Narcissistic defenses - grandiosity, idealization, and devaluation - serve to protect against overwhelming loss, past deprivations, and the realities of life (129, 145).

Anger and aggression are closely linked to narcissistic pathology, and can manifest as self-directed criticism and negativity as well as outward hostility through devaluation, criticism, and attacking behaviors toward others (78, 145, 146). These individuals struggle to acknowledge and process distressing emotions including shame, humiliation, rage, and envy (128, 132, 146). At a neurological level, narcissists experience social rejection as deeply distressing, yet they may not outwardly express it, potentially leading to reactive aggression and relational instability (96). Processing painful emotions has been shown to help narcissists reduce maladaptive behaviors while increasing resilience, self-compassion, and empathy (147). Kramer et al. (128) identified emotion processing - particularly the interplay of shame and self-compassion - as facilitating change in the treatment of narcissistic patients with comorbid depression. Improved capacity to regulate one’s emotions may support the narcissist’s development of a more cohesive self-experience (78).

Alexithymia, or difficulty identifying, describing, and expressing one’s own emotions, is strongly associated with PN, where deficits in emotion identification and metacognition contribute to maladaptive coping and emotional dysregulation (74, 140, 148). Additionally, deficits in emotional recognition - the ability to perceive and interpret emotions in others - have been observed in narcissistic patients, suggesting that impaired affective processing underlies interpersonal insensitivity (149). A mentalization-based treatment approach has been explored as a potential intervention for narcissistic individuals, with theoretical support suggesting it may enhance emotional awareness and reflective capacity to reduce maladaptive coping strategies, though direct empirical validation remains limited (74).

MDMA has shown potential in enhancing emotion regulation and tolerance. It reduces the brain’s fear-avoidance response, enabling individuals to access and reprocess overwhelming emotions that might otherwise be too distressing for narcissistic patients to confront (140). When recalling autobiographical memories, MDMA increases emotional intensity and positive affect, suggesting it could facilitate emotional processing in PN (139). Additionally, MDMA enhances positive emotional experiences while blunting negative ones, potentially reducing defensive responses and increasing emotional flexibility (79, 150). Moreover, it promotes openness and trust, helping individuals engage with difficult emotions in therapy (151).

MDMA also facilitates emotional disclosure and increases comfort in discussing personal feelings (136, 137), which could be particularly important for narcissists with alexithymia, who struggle to identify and express their emotions. It enhances affective empathy for positive emotions while impairing recognition of negative emotions, such as sadness or fear, which could potentially help narcissistic individuals tolerate and engage with emotions they would otherwise suppress (152). MDMA’s ability to reduce reactivity to negative emotional stimuli could mitigate avoidance and defensiveness in therapeutic settings, both reinforcing its relevance as an intervention targeting emotional tolerance and regulation and suggesting that MDMA-AT could help narcissistic individuals to engage with difficult emotions in therapy (151, 153).

#### 3.2.4 Sense of agency

Rooted in feelings of inferiority and powerlessness, individuals with PN tend to exhibit a strong drive for control, particularly in their interpersonal relationships (130, 146). To protect their self-image and ward off underlying insecurities, narcissistic patients frequently engage in power struggles, assuming a position of dominance and superiority and resorting to the externalization of blame (145, 154). In spite of this, many have difficulties setting goals beyond self-aggrandizement and struggle to accept the reality that achieving ambitions requires commitment, effort, and the ability to tolerate frustration (129). Brown et al. (155) found that perceived agency differentiates grandiose and vulnerable narcissism, with self-esteem mediating their relationship. While individuals exhibiting more narcissistic grandiosity tend to maintain self-esteem through agentic behaviors like self-promotion, those in more vulnerable states experience lower self-agency, contributing to diminished self-worth. Strengthening self-agency may help counteract feelings of impotence rooted in past experiences of powerlessness and helplessness.

Their need for control can manifest as a resistance to change, sometimes impeding therapeutic progress (78, 130, 145). Narcissistic patients benefit from a balance of expressive interventions that offer interpretive insights when they are receptive and supportive strategies during periods of heightened vulnerability or narcissistic injury (71). Framing interpretations as hypotheses encourages collaboration and engagement, supporting self-agency. In psychoanalytic treatment, allowing the patient to take the lead reinforces their ownership of the therapeutic process (71). Flexibility in therapy empowers the patient and ensures the therapist remains responsive to their unique needs. By facilitating open-ended dialogue, the therapist can adapt their approach to the patient’s evolving needs and readiness for support (156). Given that narcissistic functioning very frequently interferes with their ability to learn from life experiences (98), therapy provides a unique opportunity to process these in a new way, promoting insight and adaptive change (78). Therapists must balance strengthening autonomy with fostering interdependence, guiding patients toward relational reciprocity.

MDMA has demonstrated potential in enhancing self-agency through greater self-acceptance, psychological openness, and emotional awareness. Research suggests that MDMA reduces social defensiveness and increases authenticity, enabling individuals to take greater ownership of their emotions and actions (79). Kuypers (157) and Hysek et al. (81) found that MDMA enhances self-compassion and empathy and facilitates deeper self-reflection and interpersonal engagement, which may promote self-efficacy in these patients. Recent studies have shown that MDMA-AT improves self-experience in patients with PTSD, including their self-compassion and emotional regulation (6). These findings highlight the value of evaluating MDMA’s capacity to foster self-agency in narcissistic pathology.

#### 3.2.5 Therapeutic alliance

The therapeutic alliance is a well-established predictor of positive treatment outcomes across a range of psychotherapeutic modalities (158–160), including psychoanalytic psychotherapy (161) and the treatment of personality disorders such as PN (162, 163). Beyond facilitating change, the alliance itself often becomes a central focus of treatment for individuals with personality disorders, providing a relational context for addressing core interpersonal difficulties (164).

For patients with PN, forming and maintaining a strong alliance is often hindered by dismissive attachment styles, distrust toward the therapist, and excessive self-reliance (59, 129). These patients struggle with interpersonal closeness, perceiving dependency as a threat to their autonomy. As a result, they frequently oscillate between idealization and devaluation of the therapist, making the therapeutic bond unstable. These relational patterns reflect the broader interpersonal difficulties characteristic of PN. Empathic listening and validation of the patient’s suffering help regulate affect and strengthen the alliance (165), while warmth and understanding engender trust and receptivity (78). Once trust is established, narcissistic patients become more open to therapeutic challenges and alternative perspectives. Thus, the therapeutic relationship serves both as a mechanism of change - allowing patients to process emotional experiences in real time - and as a treatment goal, resulting in more adaptive relational patterns (164, 165).

A key factor in maintaining the alliance is the ability to openly address relational dynamics within the therapy. Weinberg (70) emphasizes that managing ruptures and enactments through gentle confrontation is essential for maintaining engagement in therapy, with the therapist’s availability serving as a stabilizing factor. By processing these relational patterns, within the therapeutic relationship, patients can experience corrective emotional experiences that help shift maladaptive interaction styles toward healthier, more reciprocal ones (166–168). Addressing early attachment disruptions in this relational context fosters a more cohesive self-concept, strengthens self-agency and improves interpersonal functioning (167).

Studies indicate that the therapist-patient relationship is one of the strongest predictors of treatment success across psychotherapeutic approaches, including PAT (166, 169–171). Enhancing the therapeutic alliance has significant clinical implications, particularly in modalities emphasizing relational dynamics. MDMA’s unique pharmacological properties may facilitate alliance formation by increasing compassion, empathy, and emotional engagement, which contribute to deeper therapeutic work (6, 158). Research suggests that MDMA fosters trust, openness, and social bonding, essential for a strong therapeutic alliance (79). Additionally, its prosocial effects - such as increased sociability and decreased social sensitivity - have been linked to improved therapeutic engagement (81, 88), making patients less guarded and more willing to explore painful emotional material. MDMA-AT has also been shown to improve emotional regulation and strengthen self-experience, particularly in individuals with impaired self-coherence, such as those with PN (137).

Clinical trials indicate that MDMA-AT enhances key factors necessary for a robust therapeutic alliance, including self-compassion, emotional awareness, and relational capacities (158). By facilitating authentic emotional disclosure and self-reflection, MDMA enables patients to engage more deeply in therapy (136). For patients with PN, who are prone to interpersonal defensiveness and hypersensitivity to social rejection, MDMA’s ability to reduce perception of social threat may be particularly beneficial (89). By decreasing reactivity to perceived criticism and enhancing emotional empathy (152), MDMA may help narcissistic patients tolerate vulnerability and interpersonal closeness without triggering defensiveness. Additionally, by dampening reactivity to negative emotional stimuli, MDMA may support more constructive engagement with distressing relational patterns (153). Integrating MDMA-AT with psychoanalytic principles may further establish the therapeutic relationship as a corrective emotional experience, fostering meaningful psychological change in individuals with PN (170, 171).

### 3.3 Treatment framework

The integration of psychoanalytic techniques with the unique therapeutic properties of MDMA has led to the development of a psychoanalytically informed MDMA-AT aimed at enhancing psychotherapeutic outcomes for individuals with PN. This integrated approach is currently being evaluated in an investigator-initiated trial (IIT) titled “A Pilot Study of MDMA-Assisted Therapy for the Treatment of Pathological Narcissism” (https://clinicaltrials.gov/study/NCT06565494), with study approval from the University of Washington Institutional Review Board.

**Figure 1:**
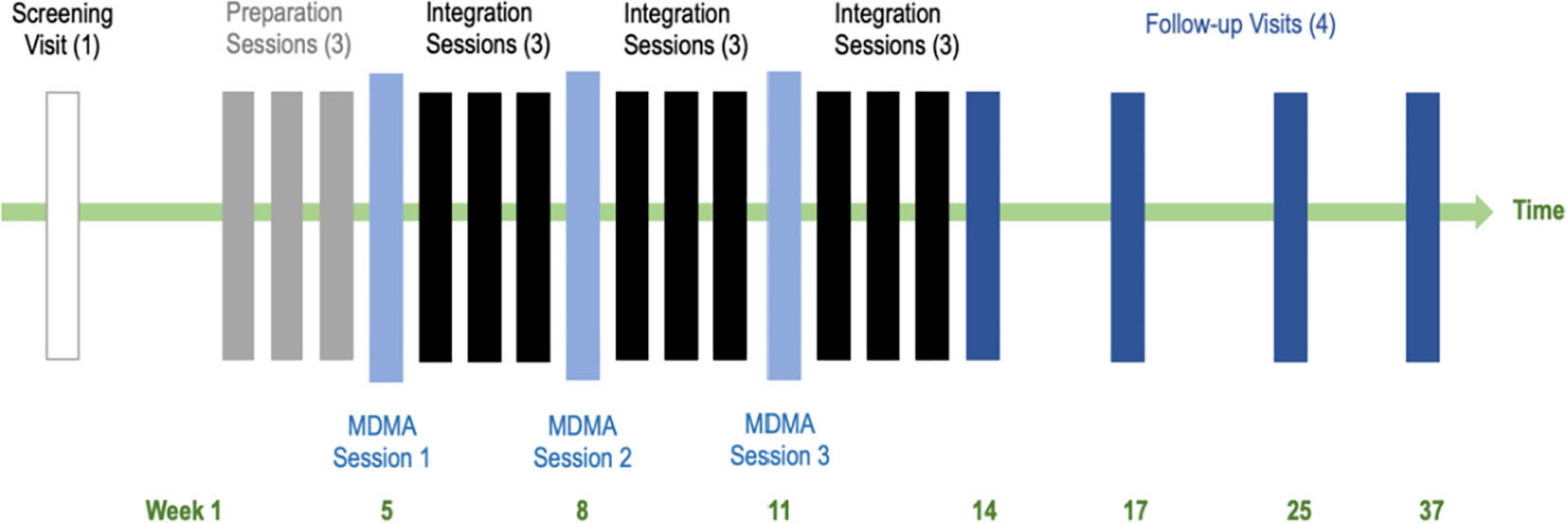
Schema of treatment model phases.

Psychoanalytic literature emphasizes the importance of a consistent therapeutic frame, particularly with narcissistic patients who often expect exceptional treatment and believe that rules and boundaries don’t apply to them. A structured therapeutic environment provides the containment and stability that a narcissist’s internal world lacks, creating a safe space for exploring overwhelming emotions and vulnerabilities. As in previous Phase 3 trials, our structured MDMA-AT approach includes both MDMA-facilitated sessions and non-drug psychotherapy sessions. This dual structure is designed to harness MDMA’s unique effects - such as reducing fear and enhancing emotional openness - to facilitate the recall and deep emotional processing of traumatic memories and feeling states without overwhelming distress. Our psychoanalytically-informed MDMA-AT model integrates this concept within the standard three-phase approach of PAT (111, 172) - Preparation, Medicine, and Integration – to provide a structured and secure space for narcissistic patients to confront and process deep-seated issues while maintaining essential therapeutic boundaries. See **Figure 1** for a schema of the treatment phases.

In our MDMA-AT model, therapists work in pairs (Therapy Team) across all three phases and play key roles in establishing a secure context – known in psychoanalytic therapy as a “holding environment” (173) and in psychedelic therapy as “set and setting” (174). Renowned psychoanalyst Winnicott (173) coined “holding environment” to describe the safe, reliable, and continuous space provided by the “good enough mother,” supporting the infant’s development through attentive care, physical holding, and emotional attunement. He extended this concept to therapy, where therapists offer a similar “holding environment,” especially for those lacking early emotional support, by providing reliability, consistency, and attunement. This environment encompasses both psychological and physical aspects, offering containment for exploring difficult emotions while maintaining appropriate boundaries. In psychedelic therapy, “set” refers to the patient’s mindset and “setting” to the environment. Therapists carefully curate both to ensure emotional readiness and a safe physical environment by building a trusting relationship, providing psychoeducation about MDMA, helping clients set intentions, selecting music, offering support during the session, and assisting with post-session Integration (174). These efforts are tailored to the patient’s unique needs and goals, emphasizing honesty, non-verbal attunement, and clear communication.

Psychoanalytic techniques known for their positive outcomes are integrated into all three phases. During the Preparation phase, a therapeutic alliance is established through open dialogue and empathic listening, exploring the patient’s history and relational patterns. Trust-building is prioritized through clarification of the patient’s expectations and intentions and preparation for the psychedelic experience. This includes discussions on safety, potential side effects, and the likelihood of encountering challenging emotions or psychological reactions, which – while their specific nature may be unpredictable – are understood as integral to the therapeutic process. The model includes three Preparation sessions, each lasting 60 to 90 minutes, before initiating any Medicine sessions. Key themes, defense mechanisms, and recurring emotional or behavioral patterns are identified, laying the foundation for meaningful insights to come from the MDMA sessions.

In the Medicine phase, the patient reclines with eye shades and listens to music to deepen the experience, while support and guidance are provided as needed throughout the approximate 6 hour MDMA session. Reflective listening and unstructured, open-ended questions encourage patients to freely explore their inner experiences, allowing unconscious material to surface and fostering deeper self-reflection and insight. MDMA reduces fear and emotional avoidance, drawing attention to previously unacceptable feelings and enabling participants to confront and experience emotions they typically avoid. Study therapists observe defenses and avoidance strategies as they emerge, helping patients remain present with challenging emotions, such as shame, contempt, envy, or vulnerability, which enhances emotional tolerance and engagement. The emotional sensitivity and openness resulting from MDMA may intensify the focus on the here-and-now therapeutic relationship, allowing transference and countertransference to surface and enabling patients to explore and potentially even modify relational patterns in real-time.

During the Integration phase, three sessions, each lasting 60 to 90 minutes, are conducted after each Medicine session to help patients process and integrate insights from their psychedelic experience. MDMA experiences are reviewed with attention to identifying recurring themes, such as disappointment and betrayal, and challenging emotions, linking them to past and current relational dynamics. Study therapists highlight ways the patient behaviorally avoids or defends against feelings, increasing the patient’s awareness of how they disconnect from difficult emotions and enabling them to engage more fully with their emotional experiences. Associations between emerging feelings and formative experiences are drawn, facilitating the processing of deeply rooted emotions and the construction of a coherent self-narrative. Study therapists also help draw connections between the therapy relationship and the patient’s other relationships, allowing participants to recognize the continuity of their relational patterns and consider more adaptive ways of interacting.

Eligible participants are aged 18-64 and must meet DSM-5 Section III criteria for PN or narcissistic personality disorder, with an awareness of their diagnosis. Before the in-person screening, participants complete a pre-screening survey featuring questions from the Personality Diagnostic Questionnaire-4+ (PDQ-4+) (175), which assesses DSM-5 Section II NPD diagnostic criteria, and the 12-item Super Brief Pathological Narcissism Inventory (SB-PNI) (176), which evaluates narcissistic grandiosity and vulnerability consistent with a diagnosis of PN. During the in-person screening, the SB-PNI is re-administered as a self-report, and the Structured Clinical Interview for the DSM-5 Alternative Model for Personality Disorders (SCID-5-AMPD) Module III (177) is conducted to confirm expressions of narcissistic grandiosity and vulnerability consistent with PN and narcissistic personality disorder.

Participants must have been engaged in individual outpatient psychotherapy with a licensed psychotherapist for at least six months and plan to continue therapy throughout the study. They must agree to discontinue psychoactive medications or substances that interact with MDMA and comply with all study requirements, including Informant assessments from their psychotherapist and a family member. Additional safety criteria include the ability to provide informed consent, complete medication washouts, and pass medical and laboratory screenings. Exclusion criteria include active suicidality within the past 12 months, co-occurring diagnoses of bipolar disorder, psychotic disorders, dissociative identity disorder, or substance use disorders. Participants with unstable medical conditions (e.g., uncontrolled hypertension, significant cardiac history, or symptomatic liver disease), pregnant or breastfeeding individuals, those with MDMA use within the past year, or those unable to adhere to study requirements are also excluded.

During the MDMA sessions, participants will receive a flexible dose of MDMA. Participants will arrive an hour before receiving their initial MDMA dose for safety checks, including health updates, vitals, and drug and pregnancy screens. The initial dose for the first session will be 80 mg, which may be increased to 120 mg in subsequent sessions based on tolerability and participant preference. A supplemental half-dose of 40 mg will be administered 1.5 to 2 hours after the initial dose unless contraindicated. This supplemental dose is designed to prolong the subjective effects of MDMA without significantly increasing physiological effects beyond those of the initial dose. The flexible dosing regimen reflects clinical practice, balances risk-benefit considerations, and accommodates the manufacturing and packaging of the capsules by the pharmaceutical company, with total doses per session ranging from 80 mg to 160 mg. Throughout the 6-hour session, the Therapy Team will monitor participants, offer support for emotional processing, and ensure safety, with discharge contingent on meeting specific physical and emotional stability criteria; participants are accompanied home and have access to overnight support.

During the approximate 12-week Treatment Period, each of the three MDMA session will be spaced approximately four weeks apart and each will be followed by three Integration sessions. The first Integration session will occur the following morning after the MDMA session and the second and third Integration sessions weekly thereafter. The primary outcome measure (Brief Pathological Narcissism Inventory, B-PNI) (178) will be obtained at the start of each of the third Integration sessions. MDMA sessions will be audio-video-recorded for safety. After the last Integration session, participants will enter the Follow-up Period lasting for approximately 6 months and comprising four endpoints: one week, one month, three months, and six months following the final Integration Session.

All Follow-up visits will be conducted in-person to collect primary and exploratory outcome self-report measures. Exploratory outcomes measures will assess changes in measures of self-esteem, reflective functioning, alexithymia, emotion regulation, self-compassion, and empathy as well as of symptomatology of common comorbidities including depression and substance use, depth of MDMA experience, and degree of working alliance between participant and Therapy Team. At the final Follow-up, qualitative data will be collected using a set of open-ended questions. Secondary outcomes will be collected in the same time periods to assess the impact of the participant’s PN on others using Informant-reports completed by the participant’s personal psychotherapist and a selected family member (176). Qualitative data will also be collected at the final Follow-up from Informants to report on changes in the quality of their relationship with the participant. Given the limited self-awareness and self-other discrepancies common in individuals with PN, Informant-reports are essential for capturing a more comprehensive understanding of their behavior and interpersonal effects (176).

Comprehensive safety measures will be in place to monitor and address psychological and physiological risks. Suicidality will be assessed weekly using the Columbia-Suicide Severity Rating Scale (C-SSRS) (179) at Baseline, during Medicine and Integration Sessions, and at Follow-up points. The Swiss Psychedelic Side Effects Inventory (SPSI) (180) will systematically evaluate adverse effects of MDMA, including severity, duration, and impact. Vital signs (heart rate, blood pressure, and temperature) will be recorded at key points during Experimental sessions, with cardiac screening having been conducted during the screening process to identify exclusionary conditions. Immediate medical evaluation and intervention, following American Heart Association guidelines (181, 182), will be provided for rare cardiovascular events, such as stroke or myocardial infarction. Participants experiencing serious medical complications will not proceed with further sessions. Psychological distress will be mitigated through thorough preparation, monitoring, and follow-up support, including care from participants’ personal psychotherapists.

Participants will be enrolled with the intention of potentially confronting and processing traumatic experiences and emotions. Informed consent will clarify that psychological distress, panic, or other unpleasant reactions are expected and may be part of the psychotherapeutic process. Distress may occur during MDMA sessions, from the onset of effects until they subside, or afterward, particularly during Integration sessions. Reactions may last minutes to several hours, with mild anxiety or low mood occasionally arising 1-3 days post-session. Proper preparation and follow-up support will help mitigate these responses. During Preparation sessions, participants will be informed that difficult emotions, such as grief, rage, fear, or panic, may arise. MDMA sessions will occur in an atmosphere of trust with close monitoring. In Integration sessions, thoughts, feelings, and memories will be explored to generate meaning, address lingering distress, and help participants apply new reflective functioning, affect tolerance, and empathy to everyday challenges. Throughout the 12-week Treatment Period, participants will have weekly contact with the Therapy Team and ongoing psychotherapy with their personal therapists for additional support. While it is unclear whether short-term improvements in symptoms of PN will translate into long-term outcomes, this treatment may catalyze continued change during participants’ ongoing psychotherapy.

The participant’s Therapy Team will be on-hand throughout the Treatment Period. All study therapists will be licensed healthcare providers with graduate-level professional training, clinical experience in psychotherapy and an active clinical license to practice independently according to state and local requirements. All will have specialized training in psychedelic therapy, including MDMA-AT. Their preparation includes studying the treatment manual, *Therapeutic Approach to MDMA-AT for Patients with Pathological Narcissism*, and a syllabus on contemporary literature about PN, as well as attending consultation sessions with a psychoanalytically-informed psychedelic study therapist. Each therapy pair will include one therapist with psychoanalytic training. A licensed medical physician will be on-call to handle emergencies and will be able to reach the site within 15 minutes. Additionally, a licensed professional authorized to manage and administer controlled substances will be on-site to dispense MDMA for each Experimental session.

### 3.4 Clinical challenges

Treating PN with MDMA-AT presents distinct clinical challenges. Key considerations include managing emotional dysregulation during and after MDMA sessions, addressing complex transference-countertransference dynamics that may rapidly emerge, navigating narcissistic traits and defenses that can impede therapeutic progress, and mitigating the risk of exacerbating rather than alleviating narcissistic traits.

PN is marked by volatile intrapsychic conflicts and emotional dysregulation, which may worsen during and after MDMA sessions. Narcissistic individuals exist in a state of persistent internal tension, relying on grandiosity to defend against feelings of inadequacy, seeking admiration while staying vigilant to criticism and rejection, and depending on external validation to sustain their self-worth, while simultaneously fearing dependency and clinging to fantasies of self-sufficiency (42, 146). These core conflicts create emotional instability that can be intensified by therapy and further heightened by MDMA. As MDMA’s effects wane, patients may become vulnerable to intense emotions such as narcissistic rage, shame, and anguish, potentially triggering suicidal impulses, even in the absence of chronic suicidality (69, 183, 184). Gabbard (69) highlights that suicide risk in narcissistic individuals can fluctuate dramatically, often in response to perceived failures, rejection, or humiliation. Given that MDMA may intensify emotional distress between sessions, monitoring these fluctuations is critical. Additionally, impulsivity and maladaptive coping mechanisms may contribute to sudden emotional shifts, reinforcing the need to help patients develop more adaptive emotion regulation skills (69).

While MDMA-AT aims to harness these internal experiences for therapeutic benefit, prioritizing safety is critical to prevent re-traumatization. Thorough screening, preparation, monitoring, and integration, support is essential for managing distress and mitigating suicide risk (17, 185). During the Preparation phase, patients should be informed of the likelihood of emotional distress and the potential for confronting traumatic memories. The Therapy Team should set clear expectations and implement safety plans - including suicidality assessments to monitor for ideation and behaviors – as well as provide additional support as needed between scheduled sessions.

The therapeutic alliance is a key protective factor against suicidality in narcissistic individuals, which can help regulate affect, reduce impulsivity, and provide corrective emotional experiences that mitigate feelings of abandonment or humiliation (69). Maintaining attunement to relational ruptures and repairing them in real time is crucial to prevent escalation to suicidal risk. Shame is a primary driver of suicidality in narcissistic patients, making it essential to approach this emotion carefully (69). Gabbard (69) recommends a non-punitive, curiosity-driven approach to help patients process shame without experiencing further humiliation, dissociation, or aggression. By fostering a secure therapeutic relationship, therapists can help reduce distress and create a contained context for emotional processing during MDMA-AT.

Therapeutic ruptures and transference dynamics are common challenges in treating PN and can significantly impact engagement in MDMA-AT. Therapists must stay attuned to the transference-countertransference dynamics that are central to treatment of PN (70, 128, 154). Working with narcissistic patients involves recognizing that their core injury of inadequate empathic attunement in childhood will inevitably manifest in the transference-countertransference dynamic as failed mirroring (feeling insufficiently validated), failed idealization (experiencing disillusionment and devaluation), and/or failed twinship (sensing a lack of similarity or connection) (51). These dynamics often materialize with narcissistic patients seeking admiration that quickly becomes expectation, idealizing the therapist only to later devalue them when their need for validation goes unmet, and treating the therapist as a passive sounding board, undermining any sense of mutuality (70, 71, 154).

Therapists frequently report experiencing hostile, criticized, and helpless countertransference reactions when working with NPD patients, often feeling devalued, disconnected, or ineffective in treatment (71, 186). Feelings of contempt or devaluation can cause therapists to doubt their competence or react defensively, while at other times, they may over-identify with the patient’s vulnerability and try to resist it through omnipotent control. Managing these dynamics requires therapists to tolerate and contain challenging projections while using their countertransference to gain insights into the patient’s internal experience (14). Tanzilli et al. (186) found that countertransference responses of frustration, anger, and disengagement are common and can threaten therapeutic continuity if not actively managed. Countertransference reactions are prevalent regardless of theoretical orientation (186). Patients with PN frequently misinterpret therapist interventions, often perceiving constructive feedback as criticism, leading to oppositional reactions or emotional withdrawal (70, 128, 154). This challenge is particularly relevant in MDMA-AT sessions, where heightened emotional intensity may amplify these reactions and cause them to be unconsciously enacted (187). Managing these transference-countertransference dynamics, is crucial for sustaining the therapeutic alliance and requires balancing empathic attunement with boundary-setting to prevent defensive withdrawal.

Since the therapeutic alliance is a key predictor of change, therapists must stay attuned to relational shifts and address ruptures as they arise (159). Directly acknowledging control dynamics, engaging in open discussions of the patient’s emotional experiences, and collaboratively exploring the therapist’s contribution to them have been shown to help stabilize the working alliance (188, 189). Given that PN patients often struggle to access and verbalize emotions, therapists must remain patient, validating, and persistent in helping them develop a greater awareness of their internal states (59, 60). Additionally, therapists must work to regulate their own countertransference responses, as therapists can unintentionally collude with maladaptive patterns (e.g., mutual idealization, competitiveness) (70). Notably, Tanzilli et al. (186) found that more experienced therapists report less intense countertransference reactions, suggesting that the ability to navigate these challenges improves with clinical experience.

Narcissistic traits and defenses can often obstruct progress in conventional treatment and even bring the treatment to an impasse, begging the question of whether these challenges will occur during MDMA-AT. Psychological rigidity, emotional reactivity, detachment, and interpersonal defensiveness frequently contribute to premature dropout or slow progress. A limited capacity to mentalize and consider alternative perspectives may leave patients without key therapeutic skills that MDMA alone may not sufficiently address. Some have speculated that narcissistic individuals could struggle with the self-reflective nature of psychedelics due to their reflective self-criticism (187). Both emotional reactivity and detachment make treatment challenging, often leading therapists to provide non-treatment interventions to avoid triggering intense negative emotions that could result in escalation or withdrawal (60, 188).

Expressions of contempt - through condescension, dismissiveness, and devaluation - combined with difficulty understanding others’ emotions, can provoke strong countertransference reactions that, if not carefully managed, may sabotage treatment. Interpersonal defensiveness can cause narcissistic patients to resist improvement, with grandiosity and pseudo-self-sufficiency making it difficult to internalize help, while fear of dependence causes them to devalue it as a way to stabilize their self-concept (59). Resistant to acknowledging others’ contributions, patients with PN may deny therapeutic change even as it occurs or attribute progress solely to the drug rather than the therapeutic relationship (59). Given the entrenched nature of these defenses and the delicate nature of therapeutic progress with these patients, additional periodic MDMA sessions may be necessary.

Lastly, there are concerns that MDMA-AT might amplify pre-existing defensive structures if not properly contained within a psychotherapeutic framework, particularly in individuals with personality vulnerabilities like narcissism (190). Narcissistic patients’ tendency to seek external validation and avoid deep emotional engagement could lead them to focus on the intensity and sensational aspects of the experience rather than its potential for genuine connection and self-reflection (70), resulting in an idealization of the medicine or viewing it as a quick fix. Additionally, the euphoric effects of MDMA could temporarily heighten feelings of superiority or mask underlying insecurities without addressing the root causes of narcissism. As a result, the therapeutic setting may become a stage that reinforces narcissistic tendencies - seeking validation, feeling special, and avoiding intimacy - instead of fostering true transformation (60). To counteract this, therapists must explicitly frame the medicine as a tool that facilitates, rather than replaces the therapeutic process, reinforcing the importance of integration and relational engagement (70). With careful framing of therapeutic intentions focused on self-exploration, personal growth, and psychoanalytic inquiry, these challenges can be mitigated to ensure the treatment addresses core issues instead of exacerbating them. By emphasizing emotional experience and deeper psychic conflicts, the therapeutic process is more likely to challenge - rather than reinforce - narcissistic defenses.

## 4 Discussion

Psychoanalytically informed MDMA-AT represents a novel approach for treating PN by combining the depth of psychoanalytic techniques with MDMA’s unique therapeutic effects. This model seeks to catalyze transformative change in individuals traditionally resistant to treatment by enhancing reflective functioning, expanding emotion regulation and tolerance, fostering personal agency, and strengthening the therapeutic alliance.

Integrating psychotherapy with psychedelics is essential for maximizing the therapeutic benefits of these medicines. This combination facilitates emotional processing, reduces symptoms, and promotes healing within a supportive, non-pathologizing therapeutic relationship. For individuals with early relational trauma and attachment deficits, MDMA-AT offers a unique opportunity to address core developmental wounds in the service of long-term psychological growth. While some debate whether psychotherapy is necessary to add to psychedelic treatments (104) - suggesting psychedelics alone may be effective - we contend that those with developmental trauma and personality pathology stand to benefit most from an integrated PAT (107). By leveraging the strengths of both modalities, this approach provides a comprehensive framework for healing and transformation.

Currently underway, our pilot study is the first known clinical trial to integrate psychoanalytic techniques with psychedelic medicine. It aims to provide preliminary insights into the safety and efficacy of MDMA for treating PN. Future research should refine this approach and evaluate its efficacy in larger, randomized controlled trials. While our focus is on MDMA-AT, we do not assume this is the only psychedelic medicine that could work synergistically with a psychoanalytically informed approach for PN. Further investigation is needed to explore alternative psychedelic-assisted frameworks. Nonetheless, we believe that a psychoanalytically informed MDMA-AT represents a compelling starting point for advancing treatment options for personality pathology rooted in attachment and developmental trauma.

## Data Availability

The original contributions presented in the study are included in the article/supplementary material. Further inquiries can be directed to the corresponding author.

## Footnotes

1 Expanding epidemiological assessments to include both grandiose and vulnerable presentations of PN would likely yield higher prevalence rates than previously reported.

2 A comprehensive historical overview of the psychoanalytic conceptualization of narcissism is beyond the scope of this paper. See Elsa Ronningstam’s *Psychoanalytic theories on narcissism and narcissistic personality* for a review that traces the development from Freud's initial concepts to contemporary theories by key figures such as Heinz Kohut and Otto Kernberg and the clinical implications for treating narcissistic personality disorder (In Campbell WK, Miller JD, editors. The Handbook of Narcissism and Narcissistic Personality Disorder. New York: John Wiley & Sons (2011). p. 41-55.).

3 Solms departs from classical psychoanalysis by revising Freud's conception of drives. He argues that drives become mental when they are felt at their source, rather than being unconscious energies that only become conscious when registered by the cortex. This view contrasts with Freud's idea that drives operate as unconscious "energies" within the mind. Solms posits that bodily demands are felt as affects at their origin; prior to this, they function only as autonomic regulatory mechanisms. This revision preserves the core psychoanalytic claim about innate needs while incorporating insights from contemporary neuroscience.
